# Neural Correlates of Appetite and Hunger-Related Evaluative Judgments

**DOI:** 10.1371/journal.pone.0006581

**Published:** 2009-08-12

**Authors:** Richard M. Piech, Jade Lewis, Caroline H. Parkinson, Adrian M. Owen, Angela C. Roberts, Paul E. Downing, John A. Parkinson

**Affiliations:** 1 School of Psychology, Bangor University, Bangor, United Kingdom; 2 MRC Cognition and Brain Sciences Unit, Cambridge, United Kingdom; 3 Department of Physiology, Development and Neuroscience, University of Cambridge, Cambridge, United Kingdom; Sapienza University of Rome, Italy

## Abstract

How much we desire a meal depends on both the constituent foods and how hungry we are, though not every meal becomes more desirable with increasing hunger. The brain therefore needs to be able to integrate hunger and meal properties to compute the correct incentive value of a meal. The present study investigated the functional role of the amygdala and the orbitofrontal cortex in mediating hunger and dish attractiveness. Furthermore, it explored neural responses to dish descriptions particularly susceptible to value-increase following fasting. We instructed participants to rate how much they wanted food menu items while they were either hungry or sated, and compared the rating differences in these states. Our results point to the representation of food value in the amygdala, and to an integration of attractiveness with hunger level in the orbitofrontal cortex. Dishes particularly desirable during hunger activated the thalamus and the insula. Our results specify the functions of evaluative structures in the context of food attractiveness, and point to a complex neural representation of dish qualities which contribute to state-dependent value.

## Introduction

Human eating behaviour is controlled by a number of factors, ranging from genetic to cultural ones [1]–[3]. For the current study, we selected two factors which have recently been the object of discussion in the context of eating motivation [4]–[7]. The first motivating factor is a person's current level of food deprivation, or ‘hunger’. Hunger within the context of the current study is a physiological need state, reflecting amongst other things blood glucose levels and stomach volume expansion [8]. Hunger in such a sense motivates individuals to seek food and eat [9].

The second factor of interest relates to the specific sensory and hedonic properties of a food item or dish in question – in particular the prospective value one attaches to a specific dish when considering it to eat. We term this factor ‘attractiveness’. Attractiveness refers to how nice a person thinks a dish would be – if one ate it. It is not the hedonic experience of pleasure or aversion due to actual taste of the food, but the level of expected appreciation of a dish based on learning, in particular an individual's experience of eating such or similar dishes. The level of anticipated attractiveness can therefore be retrieved from long-term memory via imagery, the mere observation of food cues, or by reading of its description.

Previous studies investigating the neural substrates of contributions to food intake focusing on hunger state and food properties consistently show a role of the orbitofrontal cortex (OFC) and the amygdala in those functions. Neurons in the primate caudolateral OFC [10] have been shown to respond to pleasant taste and odour stimuli [11]. Such responses can be reduced or abolished by linking a previously pleasant stimulus with an aversive event [12], or by extensive feeding of the animal with specifically that taste, or its components, creating sensory-specific satiety [13], [14]. Adaptive behaviour also requires the ability to make prospective judgments of the potential value of foods prior to consumption. This is made possible by creating associations between taste and other aspects of food, like smell or sight. Such associations then enable organisms to generate evaluation responses to those aspects [15]. Recent neuroimaging studies in humans showed that value representation in the OFC and in the amygdala [16], [17] can be demonstrated even for abstract representations of food such as the text of restaurant menu items displayed on a screen [5], [18].

The aim of the current study was to investigate how hunger and attractiveness contribute to the ‘incentive value’ of a prospective meal. Incentive value in this context expresses how desirable a particular food item, or meal, is at a given moment, or how much one wants it [19]. Our goal was to identify neural sites at which the factors hunger and attractiveness as well as their interactions are represented.

Participants completed a version of the restaurant task [18], while undergoing fMRI. They were asked to imagine being in a restaurant and were presented restaurant menu items. The task was to read each item description, to imagine it, and to rate how much one liked the dish. The rating served as an index of the current incentive value of the dish. To allow assessment of the role of the hunger factor, participants completed two otherwise identical experimental sessions, once while sated and once while hungry. This experimental design allowed us to address three questions. 1) Which brain structures respond to meal descriptions in a pattern consistent with the representation of attractiveness? 2) Which brain structures model the interaction between hunger state and attractiveness? 3) Which brain structures reflect the changing value of a particular meal across motivational state?

To answer the first question, we inspected the neural responses to different levels of attractiveness. These were operationalized by meal descriptions rated high or low by participants. A previous block-design study using positron emission tomography (PET) with a similar task identified the amygdala and the orbitofrontal cortex (OFC) as regions representing the value of menu items [18]. We used those results to form our hypotheses and neural regions of interest, but modified the design to allow clearer distinctions between conditions. Arana et al. [18] presented groups of items in blocks consisting of previously hypothesized high value or low value items. Using event-related fMRI, we were able to present single items rather than blocked groups, allowing ‘mixing’ of different attractiveness levels. We were also able to ask participants to rate each item immediately after presentation. The assignment of items to the high attractiveness or low attractiveness group would then occur based on the instant rating, rather than on a previously-expressed general preference. By the same token, the impact of the hunger factor on subjective incentive value could be assessed.

Our second question concerned the impact of the hunger factor on incentive value. Each participant completed two recordings, about one week apart, once while sated and once while hungry. This allowed us to compare the representations of attractiveness under different hunger states.

Humans rate food stimuli differently depending on how hungry they are, a change that is reflected by neural responses [20]. We wanted to explore the observation that some foods seem particularly attractive when one is hungry [21]. So whilst the previous question of this study targeted the impact of hunger on attractiveness of meals in general, i.e. it concerned the broad hunger-driven change in the difference between highly attractive and not attractive dishes, our final question addressed hunger driven change of attractiveness of single, concrete meal items. The aim of this search was to single out items whose attractiveness levels are particularly susceptible to increased (or decreased) hunger, and identify the neural activation that characterizes them. In the final analysis step, we identified items which displayed a value increase in the hungry experimental session relative to that item's value in the sated session, and compared neural activity to items whose value did not change across sessions.

## Methods

### Design

Eight volunteers (3 female; group average age of 27.9, SD = 4.1) participated in three experimental sessions. Before being recruited for our study, potential participants filled in a questionnaire containing health relevant questions and exclusion criteria. Participants with a history of eating disorders or other psychiatric or neurological conditions were excluded from the study. Participants underwent fMRI recording during two one-hour sessions, one in the hungry, and one in the sated condition. For the hungry condition, participants were instructed to not eat for 6 hours prior to the experiment. All recordings took place around 6 pm, so participants in that condition had not eaten since at least noon. These were the same participants as in a second study: ‘Neural correlates of affective influence on choice’, Piech, Lewis, Parkinson, Owen, Roberts, Downing, Parkinson (unpublished). The two recordings happened roughly one week apart, and the sequence of conditions was balanced across participants. In an initial session, participants completed an extended questionnaire indicating their food preferences. The information from it was then used to design individual menu choice options for the main experiment, which would include a variety of items, excluding items evoking negative responses like disgust. Each session consisted of three blocks of approximately 10 minutes' length. Immediately after the recording, participants reported their hunger level. Prior to the study, participants were informed about all its aspects and signed a written consent form. They were debriefed after the second session and paid for their time. The study was approved by the Psychology Research Ethics Committee at the University of Wales, Bangor.

### Task

In the ‘restaurant task’, participants were asked to imagine being in a restaurant for an evening meal. While in the scanner, they were presented food menu items on a screen (no actual food was presented). The text was back-projected on a screen and viewed through a mirror. An example of a menu item typically rated as highly palatable is: “Aromatic Crispy Duck: Duck, marinated in oriental spices, deep fried until golden and crispy, served with a Hoi Sin sauce, Chinese pancakes, spring onions, and cucumber.” An example of a menu item typically rated lower is: “Seared Spiced Plaice Steak: Plaice steak, lightly spiced, and served with a black bean salsa on top of wild rice with sautéed young spinach and sliced button mushrooms.” Participants' task was to read each menu item, to imagine what it would be like to be presented with it in a restaurant, and to indicate how much they would like an item in such a situation, using a response box held in their right hand. Participants indicated their rating of each item on a scale from 1 to 4 (4: would like it very much) using a keypad. Each session consisted of 3 scans of 7.5 minutes, and 36 menu ratings per scan. Each menu item appeared on the screen for 9 seconds. The fixation interval between item presentations varied between 1 and 3 seconds. Only the duration between item onset and response (i.e. not the entire 9 seconds) was modeled for the fMRI analysis.

### fMRI data acquisition and analysis

A 1.5 T Philips MRI scanner was used to acquire 22 T2* weighted slices per volume (5 mm slices, resulting in 3.75 mm×3.75 mm×5 mm voxel size), with a repetition time of 2.2 s. The slices were tilted by 30 degrees from the ACPC axial plane (anterior up) to reduce susceptibility artefacts. Thus the recorded volume included the entire brain volume excluding only ventral parts of the cerebellum. The first five volumes of each scan were discarded to avoid differences in T1-saturation. Pre-processing and statistical analysis were performed using BrainVoyager 2000 and BrainVoyager QX (Brain Innovation, The Netherlands). The functional images were slice-time acquisition corrected, subject motion corrected, spatially normalized to Talairach space [22], and smoothed with an 4 mm full width at half maximum Gaussian kernel. A correction for temporal autocorrelation and a temporal high pass filter of 0.01 Hz were applied. Anatomical scans were acquired during both fMRI recordings to ensure accuracy of the intersession alignment of functional data.

The events for the fMRI signal were modelled as follows. Duration always corresponded to the period from onset of the menu item to the participants' rating i.e. modelled separately for each event to allow for variations in response time. For the *attractiveness representation* analysis, items rated 4 and 3 were modelled together as high rating events, items rated 1 and 2 as low rating events. This allowed the data to be analyzed as two factors with two conditions each: hunger state (hungry, sated) and attractiveness (high, low). Both sessions were entered in one analysis using dummy variables which remained empty in the non-relevant condition (e.g. the rating-4-hungry variable had no events for the sated session). The general linear model used for the fMRI data analysis thus included 11 regressors. These were two trial types (high rating and low rating), six motion regressors, and one artefact regressor. The ratings were entered twice, for the sated and the hungry session. The motion predictors included transitions along the three axes and rotations around them. The artefact regressor was entered at points where gross head movement was detected during visual inspection. Columns of the stimulus design matrix were convolved with a canonical hemodynamic response function.

For the attractiveness *change* analysis, food items were grouped depending on their rating across sessions. Items which received a higher rating during the hungry than the sated session were modelled as ‘hunger foods’, items with the opposite pattern as ‘satiety foods’ and items with no change in rating as ‘neutral foods’. The general linear model for this analysis included 13 regressors. In contrast to the model described above, four regressors (high and low rating, hungry and sated) were replaced by six (hunger food, satiety food, no-change food, for both the hungry and sated conditions). Due to individual participant responding, the numbers of items in the conditions differed: the comparison condition with neutral foods had about twice as many events as the hunger foods condition, resulting in different error margins. In order to further characterize hunger foods and satiety foods, we asked a separate group of eight participants to rate each dish on two scales: sweetness and fatness. Each scale had three points, low, medium and high.

For the region of interest (ROI) analysis, peak coordinates were based on previous research and anatomical restrictions as indicated in the results section. Around the peak voxels, small volumes were constructed as cubes with 7 mm sides. Voxels which displayed missing signal, e.g. due to edge artefacts, were excluded from analysis. Voxel time series were z-score-normalized for each run and the signal for the events of interest was extracted for the individual ROIs and subjected to statistical higher level group random-effects analyses. The general linear model used for the attractiveness change analysis included 12 regressors. These consisted of two rating events for both hunger foods and satiety foods, one rating event for neutral foods, and the same motion and artefact regressors as above.

Additionally, unconstrained whole-brain random-effects analyses were conducted. Areas of functional activity were defined as clusters of 20 or more contiguous voxels which exceeded an uncorrected p-value of .0005. This is an arbitrary, while relatively stringent criterion. Our statistical analysis package allowed one method of accounting for multiple comparisons, the Bonferroni correction, which has been frequently described as overly conservative [e.g. 23]. None of the whole-brain calculated activations reported here survive such a correction.

The number of participants in the current study is relatively small. While many fMRI studies use larger sample sizes, random-effects analyses with as few as six subjects are permitted [24]. The given sample size may produce only low statistical power and render null-effects unreliable. We therefore focus the interpretation of our results on positive effects.

The use of fMRI provided us with advantages over previous studies conducted with PET [e.g. 5, see Introduction]. The price for these was the difficulty to interpret absolute signal levels of BOLD inherent to fMRI experiments. We therefore did not attempt to determine the main effect of fasting by comparing the sated and hungry sessions for all conditions.

## Results

### Behavioural analysis

Confirming the experimental manipulation, participants indicated higher levels of hunger after the scan in the hungry condition than after the scan in the sated condition (*t*(5) = 9.63, p<.0005).

Participants gave a rating to each displayed dish description while in the scanner. A repeated measures ANOVA with factors hunger state (sated, hungry) and rating (1–4(high)) revealed that overall, participants reported more high than low ratings (F(1,7) = 11.68, p = .011). An interaction effect showed that more high ratings were reported in the hungry condition (F(1,7) = 9.00, p = .020).

Meal rating during the experiment was paced by the participants, with an upper time limit of 12 seconds. An analysis of the response times revealed no effect for state or an interaction with state (Fs<1), indicating that rating difficulty did not differ between the sated and hungry sessions. There was a main effect for rating (F(1,7) = 6.32, p = .040). Follow-up tests showed that ‘extreme’ ratings (1 and 4) were made faster than ‘moderate’ ratings (2 and 3; t(7) = 4.24, p = .016), and that ratings defined as ‘high’ for the imaging analysis (3 and 4) were slightly faster than ‘low’ ratings (1 and 2; t(7) = 3.43, p = .033).

### fMRI analysis

#### Attractiveness representation

**Figure 1:**
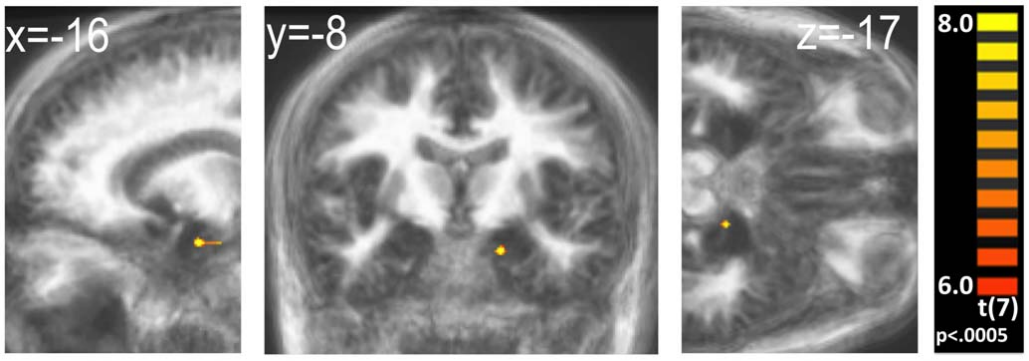
Amygdala activation during high-attractiveness trials. Sections through the amygdala for the contrast of high minus low attractiveness ratings, collapsed for both the hungry and sated conditions. The map shows t-values displayed over the averaged anatomy of all participants. No masks were used for display. Activations were defined as clusters of 20 or more contiguous voxels which exceeded an uncorrected p-value of .0005.

Studies which utilized PET to study the representation of attractiveness in a similar task [5], [18] established the amygdala and medial OFC (mOFC) as structures which responded with increased activation to groups of items indicated as highly valued by participants. In order to demonstrate that those findings reflected item-specific activity, using event-related fMRI, we compared differences between high and low attractiveness menu items (rated by the subjects as 4 or 3, and 1 or 2, respectively). The whole-brain analysis of this comparison revealed activity in the amygdala and several peaks in an area of the cerebellum (Table 1). The activated volume in the amygdala (x = −14, y = −7, z = −16, t(7) = 13.14; Figure 1), overlapped with the one reported by Arana et al. [18]. On the whole-brain level, there was no activation peak in the mOFC for this contrast.

**Table 1:** Clusters of significant activation. Inclusion criterion for the High – Low rating contrast was a p-value<.0005, and <.005 for the Hunger foods and Satiety foods.

Contrasts	right/		Coordinates			t-value	p-value
Regions	left	BA	x	y	z		
High - Low rating							
Amygdala	l		*−14*	*−7*	*−16*	13.14	<.0001
Cerebellum	r		*17*	*−40*	*−10*	8.00	<.0001
	r		*20*	*−44*	*−15*	8.34	<.0001
	r		*11*	*−47*	*−18*	8.18	<.0001
	r		*20*	*−48*	*−25*	6.93	.0002
	r		*17*	*−56*	*−21*	10.77	<.0001
Hunger foods - Neutral foods							
Dorsomedial thalamus	l		*−7*	*−14*	*6*	8.14	<.0001
Insula	r		*38*	*−9*	*3*	5.60	.0008
	r		*30*	*−18*	*21*	5.67	.0008
Lateral PFC	r	**46**	*47*	*36*	*21*	5.31	.0011
(medial frontal gyrus)							
Parietal cortex	r	**40**	*56*	*−26*	*27*	6.92	.0002
(medial occipitotemporal gyrus)							
Occipital cortex	r	**17**	*19*	*−68*	*3*	8.69	<.0001
	l	**17**	*−13*	*−70*	*6*	5.82	.0006
	r	**17**	*8*	*−96*	*3*	8.19	.0008
Satiety foods - Neutral foods							
Rostral caudate	l		*−16*	*16*	*12*	6.92	.0002
Lateral PFC	r	**8**	*13*	*29*	*51*	5.17	.0012
(superior frontal gyrus)							
Occipital cortex	r	**18**	*5*	*−77*	*−15*	5.49	.0009

Cluster extent threshold was set to 20 voxels.

#### Integration of attractiveness and hunger

**Figure 2:**
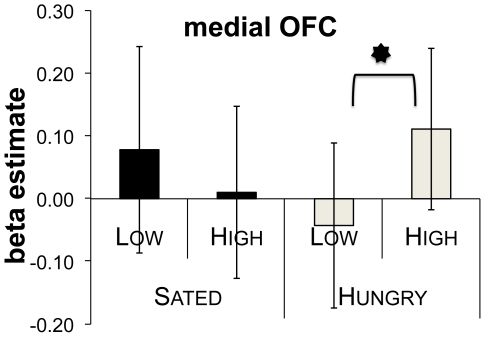
Activation in medial OFC. Z-standardized estimate of activation in an region of interest located in the medial orbitofrontal cortex (centre peak at x = −8, y = 44, z = −10), for high and low attractiveness ratings in the hungry and sated conditions. The activation shows an interaction pattern of the two factors (hunger state and attractiveness rating, F(1,7) = 8.80, p = .021). The region responds more strongly to high than to low value items during the hungry session (t(7) = 3.36, p = .012, represented by ‘*’). In the sated session, the responses do not differ (t<1.5). Error bars represent one standard error.

We performed ROI analyses of two OFC regions identified in a previous report [18]. The first, medial region (mOFC; centre at x = −8, y = 44, z = −10) showed no main effect for rating nor hunger state, (Fs<2), and a significant interaction of the two factors (F(1,7) = 8.80, p = .021; Figure 2). Follow-up t-tests revealed the following response pattern: the region discriminated between high and low attractiveness items when participants were hungry, with a higher response to high attractiveness items during the hungry session (*t*(7) = 3.36, p = .012). In the sated session, the responses did not differ (t<1.5).

The second ROI analysis examined a more lateral OFC (lOFC; centre at x = −26, y = 56, z = 0) focus from Hinton et al. [5]. Again this region revealed a significant interaction (F(1,7) = 10.76, p = .013), in the absence of main effects (*F*s<2).

#### Attractiveness change in individual items

**Figure 3:**
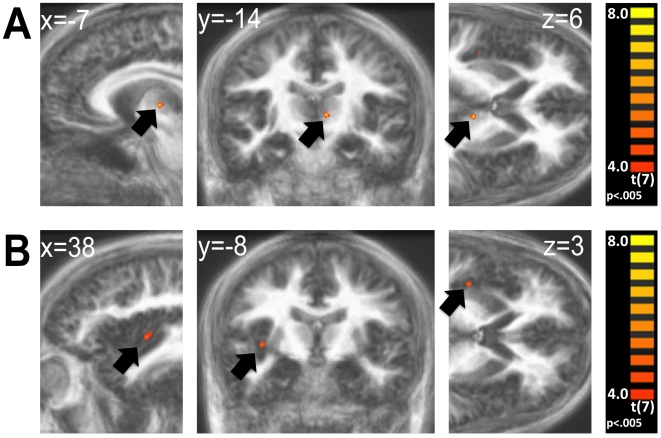
‘Hunger food’ activations. Results of the attractiveness change analysis. Significant BOLD changes in the contrast of hunger foods minus neutral foods. Panel A): Area of the left thalamus, likely in the dorsomedial nucleus. Panel B): One of two sites in the right insular cortex. Both maps are t-values displayed over the averaged anatomy of all participants. More lenient criteria were used for this analysis. No masks were used for display. Activations were defined as clusters of voxels of any size which exceeded an uncorrected p-value of .005.

The analysis of changes in food attractiveness between the sessions singled out dishes which were considered more desirable when participants were hungry (‘hunger foods’). We compared neural activity associated with these dishes with the activity associated with ‘neutral foods’, those which remained at the same rating level across the sated and hungry sessions. (Items were analysed individually per participant across sessions, and activations for each group were collapsed across the hungry and sated sessions.) Increased activity for hunger foods was found in the thalamus (a region broadly corresponding to the dorsomedial nucleus), the insula, the prefrontal, parietal, and occipital cortices (Table 1, Figure 3). We likewise identified ‘satiety foods’, dishes which showed the opposite pattern from hunger foods, i.e. were considered more desirable and rated higher when participants were sated. The whole brain contrast subtracting neutral foods from satiety foods showed increased activity in the rostral caudate nucleus, the lateral prefrontal cortex, and the occipital cortex (Table 1). It should be noted that activity peaks for ‘satiety foods’ are based on very few items (only few items were rated higher by the participants when they were sated rather than hungry) and thus are difficult to interpret.

To explain differences between hunger and satiety foods, we speculated that they may have distinct nutritional characteristics. We therefore collected ratings of sweetness and fatness of all the presented food stimuli from a separate sample of eight participants. As an indicator of the reliability, we computed the internal consistency for both scales. The consistency proved high, with Cronbach's alpha values of .94 and .89 for the sweetness and fatness scales, respectively. These ratings did not distinguish between hunger and satiety foods (all t*s*<1), however. Overall, our results show that participants' hunger influenced their food evaluation to give more items a higher incentive rating. Evaluating the descriptions of highly attractive food items strongly activated the amygdala. The activation of areas in the medial OFC for high incentive items was dependent on the participants' hunger level. When participants changed their incentive ratings of the same dish between the sated and hungry sessions, the direction of the change was reflected by activations in distinct areas of the brain. Hunger foods activated among others the thalamus and the insula, while satiety foods activated the caudate nucleus and lateral prefrontal cortex. This might indicate that dissociable neural signatures inform the valuation of food items which depends on the physiological need state.

## Discussion

The aim of the current study was to investigate how hunger and attractiveness contribute to the incentive value of a prospective meal, and at which neural sites these factors and their dynamic integration are represented. We focused on appetitive arousal induced by reading text descriptions of foods – an everyday task carried out, for example in restaurant and with cookery books. Each participant's ratings for individual meal descriptions were modelled using fMRI and to probe the representation of attractiveness, we contrasted items rated high with ones rated low on that criterion. Foremost, the contrast revealed a strong activation of the amygdala. Previous human neuroimaging and lesion studies showed that the amygdala plays a central role in the processing of affective stimuli [25], [26]. It has been shown to increase activation to appetitive stimuli such as sweet taste, pretty faces and pleasant pictures [27]–[29]. Its activation to food stimuli in general has also been shown to be modulated by hunger [30]. Our result showing increased amygdala activity for high attractiveness dishes supports its role in representing appetitive value [5], [18]. Since our investigation was aimed at representations of appetitive value, we did not include food items which would be likely to have aversive value and to elicit disgust. Therefore, our results likely reflect differences in the intensity or magnitude of value, rather than qualitative differences in appetitive foods themselves.

Several studies have looked at the neural signature in response to the evaluation of sensory stimuli such as simple tastes and smells. Anderson et al. [16] dissociated the dimensions of subjective arousal and valence [31] in the chemosensory domain within amygdala and OFC respectively. As olfaction primarily provides anticipatory signals for ingestion these data support a role for the amygdala in anticipatory value; a conclusion also arrived at by comparing cues predicting tastes, versus the ingestion those taste stimuli [17]. However, Small and colleagues [32], found a similar pattern of results to the Anderson et al. study [16] (amygdala activity for arousal and OFC activity for valence) using primary gustatory stimuli, which suggests that the amygdala may more generally code for subjective feelings of appetitive arousal induced by either anticipation or consumption. (Intuitively such signals which are likely to underlie sensations of desire or wanting would indeed be higher in anticipation of a meal.)

In contrast to fMRI research dissociating valence and arousal, it has also been argued that the amygdala codes an integrated representation of both valence and arousal – in effect a signal indicating the adaptive value of a stimulus or event [33]. Indeed, animal research supports such a role for the amygdala in the appetitive domain with processing of sensory and valence information in the basolateral subregion of the amygdala and the central nucleus underlying arousal signals [34], [35].

The current study also observed activity in the OFC for ratings of attractiveness. This medial site of the OFC responded differently to high and low levels of attractiveness, but selectively so, with significant differences in activity only observed during the experimental session when participants were hungry. In other words it showed a stronger response to high than to low attractiveness items when participants were hungry, but not when they were sated (Figure 2). Siep and colleagues [36] demonstrated similar results using food pictures, though with a different analytical approach. This suggests that this OFC area represents subjective incentive value which depends not only on the properties of the stimulus, but also on the internal state of participants. It is likely that Arana et al. [18] essentially obtained the same result in their study, but because they did not manipulate hunger, they interpreted the difference as a main effect, which truly was masking an interaction. This view is supported by a second analysis conducted in that study [18]: when they compared the signal from the amygdala and OFC with individual participant ratings, they found a significant covariation for the amygdala, but not for the OFC. In fact, Hinton and colleagues [5] included a hunger manipulation in a similar task design to that used in [18]. Their results confirm our speculations. Firstly, they do find a site in the OFC which shows a response pattern indicating an interaction of attractiveness and hunger. Our analysis of the signal from an equivalent small volume also revealed an interaction pattern for the factors attractiveness and hunger. Secondly, they too, found a main effect for attractiveness at the OFC site reported in [18]. We suspect they failed to detect an interaction at that site due to design restrictions imposed by using PET and the grouping of stimuli (we used only one meal per trial and included an immediate attractiveness rating after each trial). This is supported by the fact that the main effect is found using a whole brain analysis, not an ROI approach as in our case.

Additionally, signal extraction from the site where they [18] confirm an interaction (for both their Figure 5b and our data) reveals a telling pattern. The difference between high and low attractiveness dishes is actually reversed for the sated session, making it more detectable as an interaction than the pattern from the other site. Our claim that our results are a closer assessment of the influences of hunger state on incentive value is also supported by the fact that we actually found a behavioural effect of the hunger manipulations. The design of the current study enabled more detailed analysis of trial by trial and meal item by meal item analyses across both hungry and sated states for each participant. Of interest is the observation that our participants rated more items as highly attractive (rating 4) and fewer as not attractive (rating 1) when they were hungry, as opposed to when they were sated (Figure 2).

To summarize, our results suggest a representation of attractiveness in the amygdala, which is in agreement with its role suggested by previous human imaging studies. Our results also suggest the role of OFC in integrating attractiveness and motivational state of the individual, rather than in coding attractiveness independent of motivation. One site showing this integration pattern seems to discriminate between attractive and less attractive foods when it matters, i.e. when one is hungry, but is indifferent to them when the individual is sated. (Numerically, the pattern is reversed in the sated state, Figure 2).

The final exploration of the results shifted the focus from averaged sets of highly attractive and less attractive foods to individual dishes and how their perceived value can be altered by increased motivation in the form of hunger. We first identified which items received a higher rating when participants where hungry (‘hunger foods’) and then contrasted the neural activity they elicited with that of items which showed no rating change between the sessions (‘neutral foods’). This procedure was conducted individually for each subject. We then contrasted hunger foods with neutral foods. This contrast was conducted collapsed for both the sated and hungry session of each participant. The resulting activation map corresponds to the activation by dishes that are particularly susceptible to value increase which goes along with greater hunger. This analysis revealed activations in several regions: peaks in the thalamus, the insula, lateral prefrontal cortex, parietal cortex and occipital cortex were identified (see Table 1). Interpretation of these findings is somewhat speculative, as we only had cautious hypotheses regarding that contrast. The overall attractiveness level of the dish groups compared was not controlled for, and could have been higher for one of the groups. Keeping these issues in mind, the pattern of activity elicited by the hunger foods is nevertheless interesting and provides a starting point for further research. Above all, it appears that hunger foods are not just the attractive foods, since the activation pattern for hunger foods differed from that for high incentive foods. Neither the amygdala nor the OFC were significantly activated by hunger foods. Thus, the property which empowers them to be more desirable during the hungry state seems to be distinguishable from overall high incentive value. It is of course still possible that both structures contribute to increased value of hunger foods during hungry periods, to a degree which went undetected since we collapsed the sessions for this analysis. We speculated that it might be certain nutritional attributes which characterize a ‘hunger food’. To investigate this speculation, we asked participants (separate from the participants in the original study) to rate all the displayed food items on two scales: sweetness and fatness. Hunger foods did not show a sweetness or fatness difference from neutral foods (or satiety foods). We then modelled the fMRI data according to their sweetness and fatness ratings, and did not find differences in the regions included in the hunger foods’ imprint. Putting these observations together, it seems that neither palatability nor perceived fat or sugar content determine the incentive increase which accompanies increased hunger in some foods.

Nevertheless, some of the structures activated by hunger foods seem to be in an exceptionally good position to determine suitability of certain foods for certain states. Both the thalamus and the insular cortex have previously been shown to encode hunger signals [37]. The dorsomedial nucleus of the thalamus is closely connected to the prefrontal cortex, which allows it to coordinate hunger state and executive decision making. Activity in the insula can be found as a response to sensory properties of actual foods, particularly fat content [38], and general interoception [39]. These response properties would enable this section of the cortex to contribute to decisions combining information about specifics of food and the physiological state of the body. The activity in the parietal, occipital, and prefrontal cortices may be connected to physical differences in the appearance of the food descriptions. Whether the activity we found in the thalamus and insula indeed represents the processes we speculate on, deserves separate investigation. Such may provide a good understanding of both the neural representation and other aspects of hunger foods, and could prove valuable for research on and treatment of obesity.

The described study shows some limitations which should be addressed in future research. The ‘state’ manipulation, food deprivation of the participants, was assessed with a self-report scale, but without the use of biochemical markers, though in previous work, we found that self-reported hunger level corresponded with biochemical markers of hunger [5]. We were particularly interested in the *psychological* effects of hunger state (i.e. self-reported conscious feelings of physiological state) and consider the role of ‘feeling hungry’ on brain responses to food stimuli as valid and distinct compared to the study of actual physiological hunger-related effects.

Whilst the number of participants tested in the study was smaller than in other published work, the effect of this primarily impacts only the interpretation of null results - the absence of activations. We therefore emphasised interpretations of positive effects and the use of regions of interest in our analysis. Notwithstanding, perhaps surprising in our results may seem the absence of activations of the ventral striatum for either the attractiveness contrast or for hunger foods. This is because the ventral striatum has been widely reported to be activated by reward expectation or during reward delay [for reviews see 40], [41]. The critical distinction, and important nature of our results, is that participants *evaluated* the incentive value of menu items, knowing they would not get to eat the food. Hence, we were unlikely to observe reward expectation.

A potential problem that arises from utilizing ROIs based on PET data in an fMRI study is the following. FMRI data are prone to showing vein draining effects, leading to displaying activity ‘downstream’ of the actual site. Another problem, specific to OFC recordings, are distortion effects due to proximity of cavities. We addressed both issues by choosing ROIs that are large enough to ‘enclose’ these effects (each ROI had a volume of>300 mm^3^).

Overall, the results of our study show that the amygdala represents incentive value in written descriptions of affectively relevant stimuli. We demonstrate that areas of the orbitofrontal cortex respond to the overall attractiveness of dishes as a function of motivational state. We also suggest the thalamus and insula as structures that potentially help to choose the right food items at the right time.
